# Panax ginseng alleviates diabetic peripheral neuropathy by suppressing mitochondrial dysfunction and oxidative stress via modulation of the RAGE, NF-κB, and Nrf2 pathways

**DOI:** 10.1016/j.jgr.2025.09.003

**Published:** 2025-09-15

**Authors:** Pengcheng Liu, Teng Yang, Jie Zhang, Bang Su, Qidong Shi, Yao Song, Xin Yu

**Affiliations:** a Department of Hand and Foot Surgery, Orthopaedics Clinic, The First Hospital of Jilin University, China; b Jilin Province Key Laboratory of Tissue Repair, Reconstruction and Regeneration, China; c Department of Orthopedics, Lequn Branch, The First Hospital of Jilin University, China; d Department of Cardiovascular Medicine, The First Hospital of Jilin University, China; e Department of Operating Room, Lequn Branch, The First Hospital of Jilin University, China

**Keywords:** *Panax ginseng*, Diabetic peripheral neuropathy, Oxidative stress, Mitochondrial dysfunction, Network pharmacology

## Abstract

**Background:**

Diabetic peripheral neuropathy (DPN) represents a prevalent complication associated with diabetes mellitus, characterized by progressive nerve degeneration that leads to chronic pain and sensory dysfunction. Existing treatment options are inadequate in addressing the multifaceted underlying mechanisms of DPN, underscoring the necessity for the development of novel multitarget therapeutic strategies.

**Methods:**

A systematic evaluation explored *Panax ginseng*'s (GS) therapeutic efficacy using a multidisciplinary approach, administering the extract to diabetic rats for nerve assessments and conducting in vitro tests on Schwann cells and ND7/23 neuron cells under high glucose. Network pharmacology and molecular docking identified key targets and pathways, validated through experiments on mitochondrial function, oxidative stress, inflammation, and apoptosis.

**Results:**

Administration of GS significantly improved motor nerve conduction velocity, increased pain thresholds, and restored myelination in DPN rats. In vitro, GS enhanced RSC96 and ND7/23 cell viability and migration. Network pharmacology indicated GS modulates RAGE/NF-κB and Nrf2/PPARγ pathways, reducing oxidative stress, enhancing mitochondrial function, and lowering inflammatory cytokines. It also normalizes the Bcl2/Bax ratio to mitigate apoptosis.

**Conclusion:**

The findings of this study illustrate that GS mitigates DPN through a synergistic modulation of mitochondrial function, oxidative stress, neuroinflammation, and apoptosis pathways, with particularly significant effects on maintaining Schwann cell and Neuron cell functionality. Our results provide mechanistic insights that advocate for the repurposing of whole GS extract as a multitarget therapeutic agent for managing diabetic complications.

## Graphical abstract

**Figure:**
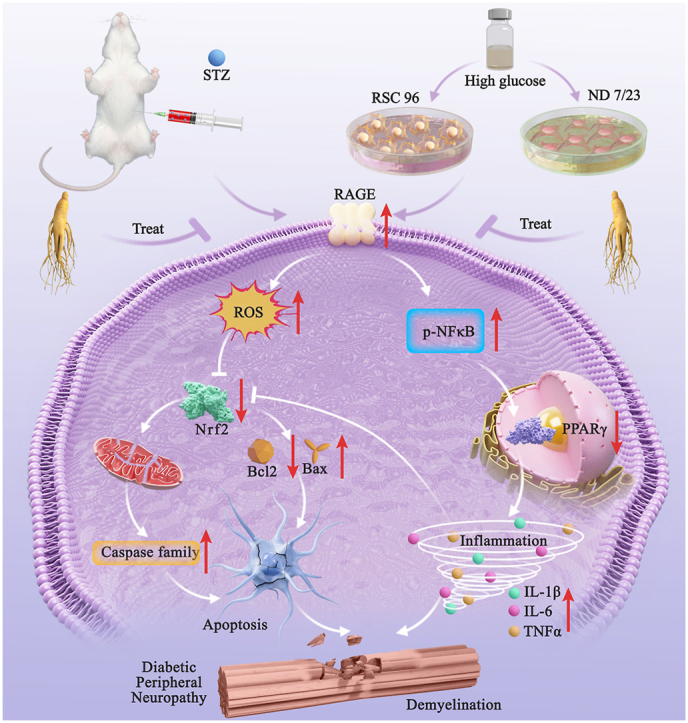

## 1 Introduction

Diabetes mellitus has become one of the most common endocrine disorders worldwide. Among its various complications, diabetic peripheral neuropathy (DPN) is the most frequently encountered, affecting about 50 % of people with diabetes. This condition is closely linked to prolonged disease duration and poor glycemic control, which significantly reduces both quality of life and overall prognosis [1]. Clinically, DPN presents with symptoms such as numbness, tingling, and pain in the extremities. In more severe cases, it can lead to motor impairment and, in extreme situations, may require amputation [2]. As a result, developing effective treatment strategies to alleviate the symptoms of DPN has become a crucial focus in clinical research.

The causes of DPN are intricate and involve multiple factors, with chronic high blood glucose levels being a key trigger that leads to inflammation, oxidative stress, and the death of nerve cells [3]. In people with diabetes, there is often a persistent state of low-grade inflammation, marked by increased levels of pro-inflammatory substances like tumor necrosis factor-alpha (TNF-α) and interleukin-6 (IL-6). These substances significantly contribute to nerve injury and dysfunction. Additionally, there is an increase in oxidative stress within the nerve tissues of diabetics, which is indicated by higher levels of lipid peroxidation and damaged DNA [1]. This oxidative stress is closely associated with the progression of neuropathy and activates various signaling pathways, such as the protein kinase C (PKC) and NF-κB pathways, which further enhance nerve cell death and impair their function [4]. Moreover, both high blood glucose and oxidative stress lead to the increased expression of genes related to apoptosis, especially those in the caspase family, like caspase-3 and caspase-9, which are vital for regulating programmed cell death [5]. Despite the availability of some treatments, there is still a significant gap in the development of effective multi-target therapies for DPN, underscoring the urgent need for new and innovative treatment approaches.

The therapeutic potential of traditional Chinese medicine in managing diabetes and its related complications is gaining significant attention. Among the various herbal remedies, *Panax ginseng* (GS) is particularly notable due to its rich array of bioactive substances, such as ginsenosides, polysaccharides, and aromatic compounds. These compounds are recognized for their anti-inflammatory, antioxidant, and neuroprotective properties [4,6,7]. For example, ginsenoside Rb1 has been shown to activate the PI3K/Akt signaling pathway, which enhances neuronal survival and reduces apoptosis. On the other hand, ginsenoside Rg3 is known to inhibit the release of inflammatory cytokines, contributing to a decrease in neural inflammation. However, the exact mechanisms through which GS exerts its therapeutic effects on DPN are still not fully understood. Network pharmacology offers an innovative approach to thoroughly investigate the interactions among various components and targets involved in complex diseases by creating networks that connect drugs, targets, and diseases [8]. This methodology is particularly promising for exploring GS, as it can address multiple aspects of the intricate pathology associated with DPN.

This investigation aims to explore the therapeutic effectiveness of GS in treating DPN using a comprehensive approach. First, we will assess how GS improves peripheral nerve function in rat models of DPN induced by streptozotocin, focusing on both functional and structural analyses. Following this, we will conduct in vitro experiments to evaluate GS's ability to enhance cell viability and migration in hyperglycemic conditions. Additionally, we will utilize network pharmacology to identify key targets and pathways linked to the active components of GS in the treatment of DPN, which will be validated through molecular docking and experimental methods. We will also investigate how GS reduces oxidative stress, possesses anti-inflammatory effects, and prevents cell death, thereby clarifying its multi-target mechanisms in the context of DPN. This research represents an essential step toward clinical applications, suggesting a new therapeutic strategy for managing DPN.

## 2 Materials and methods

### 2.1 Reagents and antibodies

The six-year-old main roots of Panax ginseng C.A. Meyer were collected from Wudu County, Gansu Province, China. A voucher specimen (PG2023-018) has been deposited at the Changchun University of Chinese Medicine. It was generously supplied and authenticated through pharmacognostic analysis by Professor Dandan Wang from the same institution. The High-Performance Liquid Chromatography (HPLC) profile of the primary constituents of GS has been documented in prior studies [9]. 200 g of *Panax ginseng* root (powdered to 40 mesh) was extracted twice by refluxing with 1600 ml of distilled water at 100 °C for 1.5 h each time. The filtrates were combined and concentrated under reduced pressure at 60 °C to obtain a thick extract. The concentrate was pre-frozen at −30 °C for 20 min and then lyophilized using a freeze-dryer to obtain a solid powder. Finally, 58 g of *Panax ginseng* extract powder was obtained.

Antibodies directed against MBP, NF-H, RAGE, Nrf2, PPARγ, p-IκB, NFκB, p-NFKB, β-Actin, IL-1β, IL-6, TNF-α, C-casp3, Bax, and Bcl2 were procured from Wuhan Sanying Biotechnology Co., Ltd. located in Wuhan, China.

### 2.2 Experimental animals and ethical considerations

Male Sprague-Dawley rats (200–250g) were obtained from Liaoning Changsheng Biotechnology, housed under a 12-h light/dark cycle, with free water access and welfare measures in place. Animal models were established and maintained by Suzhou Weiyuan Biotechnology Co., Ltd. Park Branch (Suzhou, China) under standard laboratory conditions. The animal experiments in this study were approved by the Institutional Animal Care and Use Committee (IACUC) of Suzhou Weiyuan Biotechnology Co., Ltd. Park Branch (Approval No. IACUC-20240824).

### 2.3 DPN model and pharmacological interventions

Diabetes was induced in subjects with 60 mg/kg streptozotocin (STZ) in citrate buffer via injection after fasting. Hyperglycemia was confirmed three days later using a glucose meter (Roche, Basel, Switzerland), categorizing rats with blood glucose ≥16.7 mmol/L as diabetic. Blood glucose was measured at 4 and 8 weeks; those above 16.7 mmol/L at 8 weeks were identified as DPN rats and treated with GS. The GS extract was suspended in sterile physiological saline (0.9 % NaCl), with fresh preparation before each administration; oral gavage was performed daily at a standard volume of 5 ml/kg for 8 weeks. Diabetic rats were divided into five groups (n = 6): control, DPN, and three GS treatment groups receiving low (100 mg/kg/d), medium (150 mg/kg/d) and high (200 mg/kg/d) doses.

### 2.4 Functional assessment of peripheral nerves

#### 2.4.1 Assessment of mechanical pain threshold and thermal response latency

To assess rat neurological function, we measured mechanical pain threshold and thermal response latency every four weeks using a Von Frey Pain Measurement Instrument (IITC Life Science Inc., Woodland Hills, CA, USA) and a hot plate apparatus (UGO Basile Inc., Varese, Italy), following established methodologies [10,11].

#### 2.4.2 Measurement of motor nerve conduction velocity (MNCV)

After 8 weeks of treatment, MNCV was assessed using an electromyography machine (Keypoint, Meridian, Neurolite AG, Switzerland), recording latency by stimulating the sciatic nerve at the notch and ankle, measuring the distance, and calculating MNCV using the formula: MNCV (m/s) = d/t [10].

### 2.5 Histological assessment of peripheral nerves

#### 2.5.1 Toluidine blue staining

As previously documented, axons exhibiting atypical myelination display distinct features such as demyelination, axonal degeneration, disorganized myelin sheaths, and the presence of myelin vacuoles [12]. We performed a manual enumeration of the abnormally myelinated axons and presented the findings as a ratio of abnormal axons to the total axon count.

#### 2.5.2 Transmission electron microscopy (TEM)

The ultrastructural features of myelin sheaths were analyzed using Transmission Electron Microscopy (TEM) (JEOL, Japan) following established methods [12]. Key parameters included axon and myelin sheath diameters and the G-ratio (axon diameter/myelin diameter), providing an objective assessment of myelin and axon injuries.

#### 2.5.3 Immunofluorescence assay

Nerve tissue sections were permeabilized with 0.5 % Triton X-100 for 10 min, incubated with 10 % fetal bovine serum for 2 h, then treated with primary antibodies NF-H and MBP (1:200) overnight at 4 °C. After three PBS washes, secondary antibodies AF488 and AF594 (1:1000, Thermo Fisher Scientific, USA) were applied for 2 h.

### 2.6 Cell culture and treatment

The RSC96 and ND7/23 cell lines were obtained from the Institute of Basic Medical Sciences in Beijing and maintained in DMEM with 10 % FBS at 37 °C and 5 % CO2. A glucose concentration of 100 mM was used to simulate high glucose conditions [13]. GS extract was reconstituted in DMEM at 25 μg/ml and 100 μg/ml, and cells were treated with GS and high glucose for 48 h, divided into four groups: normal glucose (CON), high glucose(100 mM, HG), high glucose with GS at 25 μg/ml, and high glucose with GS at 100 μg/ml.

#### 2.6.1 Cell viability assay

Cell viability of RSC96 and ND7/23 cells was assessed using a CCK-8 assay, and apoptotic cells were identified with an Annexin V-FITC and PI co-staining kit (Beyotime, China). Cells were harvested, resuspended in binding buffer at 500 cells/mL, and incubated with 5 μL of Annexin V-FITC and PI for 5 min in the dark. Samples were analyzed with a BD flow cytometer (BD Biosciences Inc., San Diego, CA, USA) and FlowJo software (version 10; Ashland, OR, USA).

#### 2.6.2 Live/dead cell staining

The Calcein-AM/PI kit (Beyotime, China) assessed live and dead cells by co-incubating RSC96 and ND7/23 cells with a 1 μM Calcein-AM and 3 μM PI solution for 30 min, followed by imaging with fluorescence microscopy (Olympus, Japan).

#### 2.6.3 Wound healing assay

RSC96 cells were seeded in 6-well plates at 5 × 10^5^ cells per well in four groups. A sterile pipette tip created a scratch in the cell monolayer, and detached cells were removed by rinsing with PBS. Wound areas were photographed at 0, 24, and 48 h. Cell motility was calculated using the formula: Cell motility (%) = (1 - (Wound width at 24 or 48 h/Wound width at 0 h)) × 100 %.

#### 2.6.4 Measurement of oxidative stress, reactive oxygen species (ROS), and mitochondrial membrane potential (MMP)

After exposing RSC96 and ND7/23 cells to high glucose and treating with GS for 48 h, protein concentration was measured using a BCA kit, Malondialdehyde (MDA) (cat. no. S0131S; Beyotime Institute of Biotechnology) levels and superoxide dismutase (SOD) (cat. no. A001-3-2; Nanjing Jiancheng Bioengineering Institute) activity were assessed per manufacturer protocols, ROS levels were evaluated with a Reactive Oxygen Species Kit (Solarbio, cat. no. CA1410, Beijing, China), and MMP was determined using a JC-10 Assay Kit (Solarbio, cat. no. CA1310, Beijing, China) by calculating the fluorescence intensity ratio of red (aggregated state) to green (monomeric state).

#### 2.6.5 Enzyme-linked immunosorbent assay (ELISA)

Supernatants from RSC96 cells in four groups were collected, and IL-1β, TNF-α, and IL-6 concentrations were measured using a rat ELISA kit per manufacturer protocols (Wuhan Sanying Biotechnology Co., Ltd., Wuhan, China).

### 2.7 RT-qPCR

The expression levels of IL-6, TNFα, and IL-1β were assessed through RT-qPCR. Total RNA was extracted from RSC96 cells utilizing the Trizol kit (BioTeke, CHN) in accordance with the supplied instructions. Reverse transcription for each RNA sample was performed using a reverse transcription kit (BioTeke, CHN) to synthesize the corresponding cDNA. Quantitative PCR was executed to measure the RNA levels of the target genes employing SYBR Green I dye (Invitrogen). GAPDH served as the internal control. Each sample underwent a minimum of three independent assessments, and the results were averaged to ensure precise analysis. The primer sequences employed for RT-qPCR can be found in Supplementary Table S1.

### 2.8 Western-blot (WB) assay

20 μg of protein were resolved by 10 % SDS-PAGE and subsequently transferred onto PVDF membranes (Millipore, Bedford, MA, USA). Following the blocking of the membrane with 5 % BSA, it was incubated overnight with primary antibodies at 4 °C. The next day, the membrane was exposed to a peroxidase-conjugated rabbit secondary antibody. Densitometric analysis of the bands was performed and normalized to β-Actin utilizing ImageJ software.

### 2.9 Network pharmacology analysis

We used the TCMSP (https://tcmsp-e.com/) to find Panax ginseng components with at least 30 % OB and 0.18 DL, then cross-referenced their targets with diabetic neuropathy-related targets from GeneCards (https://www.genecards.org/), OMIM (https://www.omim.org/), and DisGeNET (https://www.disgenet.org/). A Venn diagram showed common genes, and we created a network linking drugs, components, and targets using Cytoscape 3.7.2. The intersecting genes were uploaded to STRING (https://string-db.org/) to form a protein-protein interaction (PPI) network, where node size and color indicated connectivity, and edge thickness represented combined scores, helping identify core targets. We submitted intersecting drug and disease-related genes to the DAVID (https://david.ncifcrf.gov/summary.jsp) database for functional annotation, focusing on Biological Process, Cellular Component, and Molecular Function to clarify GS's target proteins in DPN treatment. We conducted KEGG pathway enrichment analysis on relevant signaling pathways, selecting the top 10 enriched terms from each GO category and identifying 20 significant KEGG pathways associated with DPN (P < 0.01) as key processes for GS's therapeutic effects. Finally, molecular docking studies using AutoDock Vina (version 1.1.2) validated the binding interactions between active components and key targets.

### 2.10 Overexpression of RAGE

The RAGE overexpression vector (oe-RAGE) and empty control vector (oe-Null) were designed and constructed by Shanghai Sangon Biotech Co., Ltd. (Shanghai, China). Detailed vector sequences and construction methods followed previously published protocols [14].

### 2.11 Statistical analysis

The image processing involved in the current study was conducted using Image J (NIH, USA). All data were analyzed using GraphPad Prism (GraphPad Software, USA). Data that conformed to a normal distribution were subjected to further analysis via One-way ANOVA, while the Wilcoxon test was applied to data that did not meet normal distribution criteria. Results are presented as mean ± standard deviation (M±SD), where *P* < 0.05 was considered statistically significant (ns: *P* > 0.05, ∗: *P* < 0.05, ∗∗: *P* < 0.01; ∗∗∗: *P* < 0.001; ∗∗∗∗: *P* < 0.0001).

## 3 Results

### 3.1 GS mitigated neuropathic characteristics in DPN rats

**Fig. 1:**
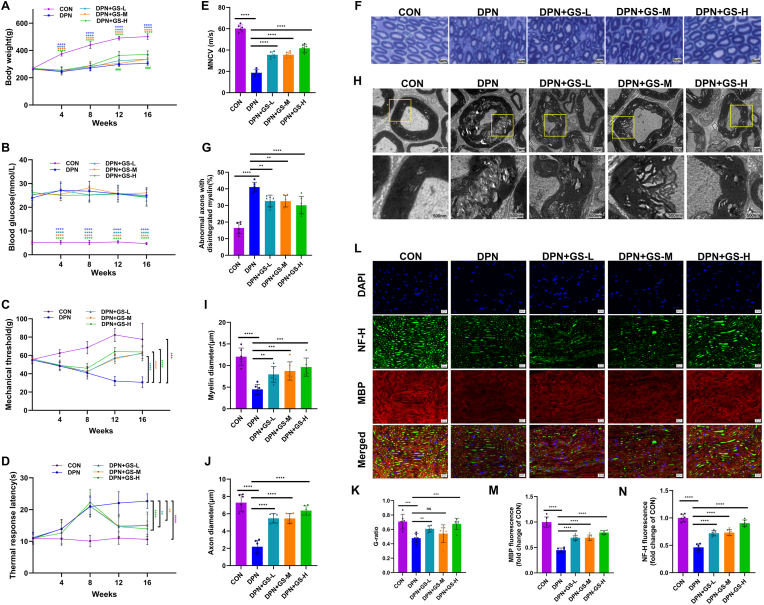
***Panax ginseng* improves peripheral nerve function and morphology in DPN rats**. **(A)** Body weight changes in five groups (n = 6). **(B)** Blood glucose levels in five groups (n = 6). **(C)** Mechanical pain thresholds assessed by Von Frey filaments (n = 6). **(D)** Thermal response latency measured via hot plate test (n = 6). **(E)** Motor nerve conduction velocity (MNCV) in sciatic nerves (n = 6). **(F)** Representative toluidine blue staining images (200 × ; n = 6) of sciatic nerves. **(G)** Quantification of abnormal axons. **(H)** Representative TEM images (20000 × ; n = 6) of sciatic nerves. **(I)** Quantification of myelin diameter (n = 6). **(J)** Quantification of axon diameter (n = 6). **(K)** Quantification of G-ratio (n = 6). **(L)** Representative immunofluorescence staining images for MBP and NF-H of sciatic nerves (400 × ; n = 6). **(M)** Quantification of fluorescence intensity of MBP (n = 6). **(N)** Quantification of fluorescence intensity of NF-H (n = 6). The results are indicated by mean ± SD. ∗*P* < 0.05, ∗∗*P* < 0.01, ∗∗∗*P* < 0.001, ###*P* < 0.001, ∗∗∗∗*P* < 0.0001.

We investigated GS's neuroprotective effects in DPN rats, where STZ-induced diabetes led to weight loss and increased blood glucose (Fig. 1A and B). GS treatment for eight weeks slightly increased diabetic rats' weight but remained lower than healthy rats. Despite persistent hyperglycemia, GS significantly improved mechanical pain thresholds (Fig. 1C) and thermal response latency (Fig. 1D). Electrophysiological tests showed increased MNCV in the GS group compared to the untreated group (Fig. 1E). However, there was no significant difference between the GS-L and GS-M groups.

### 3.2 GS mitigates morphological changes in peripheral nerves of DPN rats

Toluidine blue staining showed well-preserved sciatic nerves in controls, while DPN rats had demyelination and axonal shrinkage (Fig. 1F). TEM revealed clear myelin in controls but indistinct in DPN group. DPN rats had vacuolar lesions and axonal atrophy, which improved with GS treatment (Fig. 1H). Morphometric analysis indicated increased abnormal axons in DPN, reduced after GS treatment (Fig. 1G). Untreated DPN rats had decreased G-ratio (Fig. 1K) and myelin/axon diameters (Fig. 1I and J) compared to controls, but GS improved these changes, suggesting protective effects against DPN.

### 3.3 GS reverses the reduction of myelin and axonal protein expression in DPN rats

The myelin sheath encases axons in the peripheral nervous system, and DPN leads to myelin loss and axon shrinkage [15]. Myelin basic protein (MBP) is crucial for myelination [16,17], while neurofilament heavy chain (NF-H) indicates axonal atrophy [18]. We examined MBP and NF-H expression in the sciatic nerve using immunofluorescence, finding MBP in the myelin sheath and NF-H in axons (Fig. 1L). In DPN rats, both MBP and NF-H levels decreased, indicating demyelination and impaired regeneration (Fig. 1M and N). Treatment with GS increased MBP and NF-H levels, suggesting it may aid myelin and axon repair in DPN.

### 3.4 GS provides protection to RSC96 and ND7/23 cells against damage induced by hyperglycemia

**Fig. 2:**
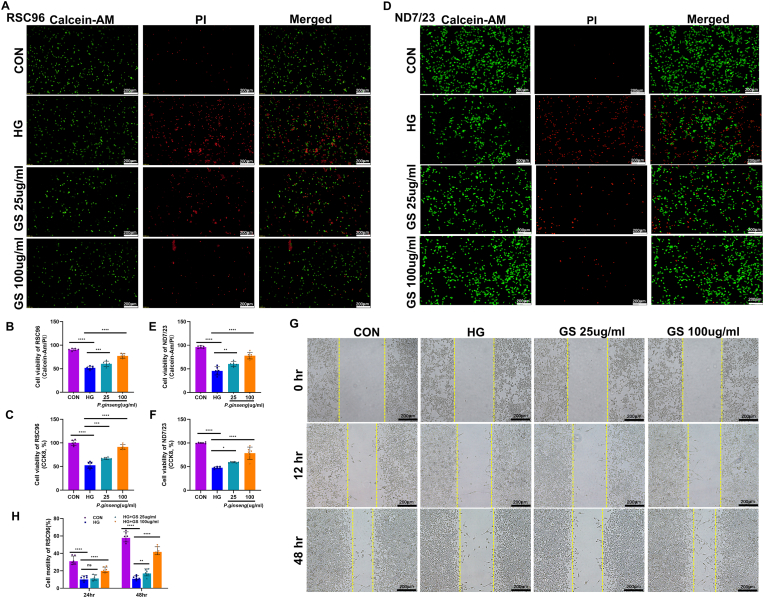
***Panax ginseng* protects RSC96 and ND7/23 cells against high glucose-induced damage in vitro. (A)** Representative fluorescence images for live/dead cell staining of RSC96 cells using Calcein-AM and PI (40 × ; n = 6). **(B)** Quantitative analysis of live/dead cell staining for RSC96 (40 × ; n = 6). **(C)** Cell viability of RSC96 cells assessed by CCK-8 assay (n = 6). **(D)** Representative fluorescence images for live/dead cell staining of ND7/23 cells using Calcein-AM and PI (40 × ; n = 6). **(E)** Quantitative analysis of live/dead cell staining for ND7/23 (40 × ; n = 6). **(F)** Cell viability of ND7/23 cells assessed by CCK-8 assay (n = 6). **(G)** Representative images for wound healing assay of RSC96 cells (40 × ; n = 6). **(H)** Quantitative analysis of cell motility (n = 6). The results are indicated by mean ± SD. ∗*P* < 0.05, ∗∗*P* < 0.01, ∗∗∗*P* < 0.001, ∗∗∗∗*P* < 0.0001.

We examined GS's protective effects on RSC96 cells under high glucose (100 mM), finding decreased cell viability, which GS treatment alleviated (Fig. 2C). Live/dead staining revealed increased dead cells with high glucose (Fig. 2A and B), while GS reduced cell death and improved viability. In ND7/23 neuronal cells, the same results as those in RSC96 Schwann cells were observed. GS effectively ameliorated high glucose-induced neuronal cell damage and enhanced cell viability (Fig. 2D, E, F). Additionally, GS accelerated wound closure in scratch assays (Fig. 2G and H), indicating it counteracts high glucose's negative impact on cell migration.

### 3.5 Network pharmacology reveals multi-target mechanisms of GS in DPN

We screened the TCMSP database, identifying 17 active constituents of GS and 114 therapeutic targets (Supplementary Table S2). After removing duplicates, we found 6374 targets related to DPN (Supplementary Table S3), with 83 common targets for GS in treating DPN (Fig. 3A, Supplementary Table S4). Analyzed in the STRING database, key targets included TNF, AKT1, IL-1β, PTGS2, PPARγ, BCL2, CASP3, and JUN (Fig. 3B). The drug-component-target network revealed five crucial components: beta-sitosterol, kaempferol, stigmasterol, fumarine, and frutinone A (Fig. 3C). The GO functional enrichment analysis revealed that GS significantly influences biological processes relevant to DPN treatment, including responses to xenobiotic stimuli, positive regulation of apoptotic processes, and positive regulation of gene expression; cellular components such as the plasma membrane, cytosol, and membranes; and molecular functions including protein binding, identical protein binding, and enzyme binding (Fig. 3D). Additionally, the KEGG pathway analysis clarified essential pathways involved in the therapeutic effects of GS on DPN, such as the AGE-RAGE signaling pathway in diabetic complications, TNF signaling pathway, C-type lectin receptor signaling pathway, IL-17 signaling pathway, Th17 cell differentiation, apoptosis, and the NF-κB signaling pathway (Fig. 3E).

**Figigure3:**
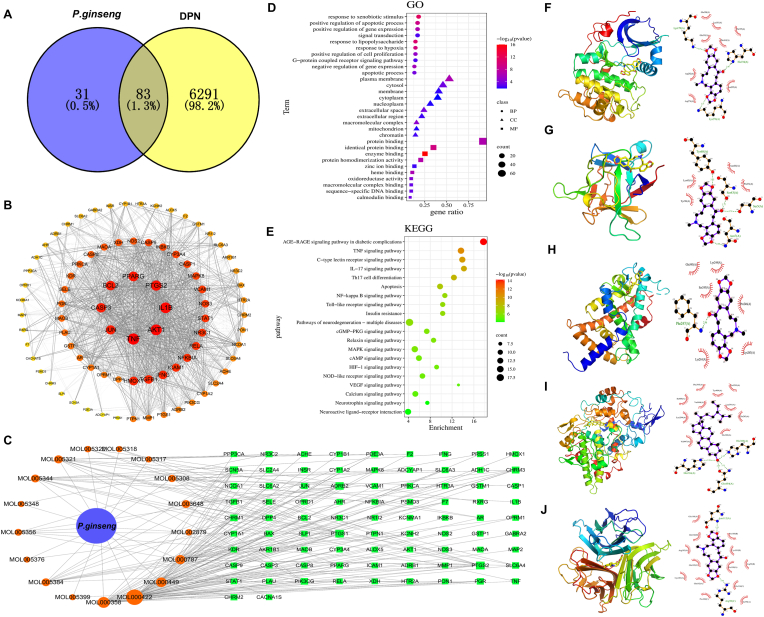
**Network pharmacology and molecular docking reveal the multi-target mechanisms of *Panax ginseng* in DPN**. **(A)** Venn diagram showing the intersection between *Panax ginseng* and DPN. **(B)** Protein-protein interaction (PPI) network of the 83 overlapping targets. **(C)** Drug-component-target network constructed using Cytoscape. **(D)** GO functional enrichment analysis of the 83 overlapping targets. **(E)** KEGG pathway enrichment analysis. **(F)** Molecular docking validation of fumarine-AKT1. **(G)** Molecular docking validation of fumarine-IL-1β. **(H)** Molecular docking validation of fumarine-PPARγ. **(I)** Molecular docking validation of beta-sitosterol-PTGS2. **(J)** Molecular docking validation of fumarine-TNF.

### 3.6 Molecular docking

We identified five targets and compounds for semi-flexible molecular docking, with binding energy values indicating spontaneous interactions. Each small molecule effectively interacted with target proteins' active sites. Fumarine formed hydrogen bonds with AKT1 (Lys276, Lys179, His194) (Fig. 3F), IL-1β (Asn7, Ser5, Ser43, Tyr68) (Fig. 3G), PPARγ (Phe247) (Fig. 3H), and TNF (Arg98, Asn112) (Fig. 3J). Beta-sitosterol formed bonds with PTGS2 (His214, Gln454, His386) (Fig. 3I). These molecules also showed significant hydrophobic interactions. Detailed results are in Supplementary Table S5.

### 3.7 GS mitigates high glucose-induced activation of the RAGE/NF-κB pathway and augments Nrf2/PPARγ signaling

**Fig. 4:**
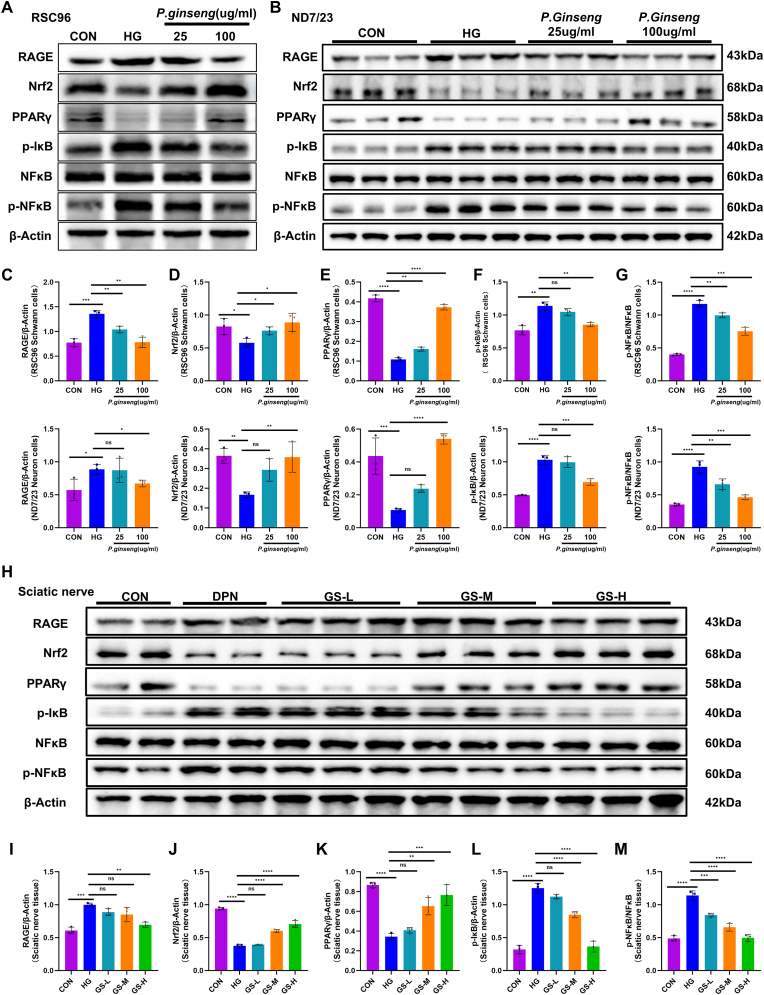
***Panax ginseng* modulates the RAGE/NF-κB/Nrf2 signaling axis in high glucose-treated cells and DPN sciatic nerves**. **(A)** Western blot analysis of key proteins in the RAGE/NF-κB/Nrf2 pathway in RSC96 cells (n = 3). **(B)** Western blot analysis of key proteins in the RAGE/NF-κB/Nrf2 pathway in ND7/23 cells (n = 3). **(C)** Quantification of RAGE expression. **(D)** Quantification of Nrf2 expression. **(E)** Quantification of PPARγ expression. **(F)** Quantification of p-IκB expression. **(G)** Quantification of p-NF-κB/NF-κB ratios. **(H)** Western blot analysis of key proteins in the RAGE/NF-κB/Nrf2 pathway in sciatic nerves of DPN rats (n = 3). **(i)** Quantification of RAGE expression. **(j)** Quantification of Nrf2 expression. **(k)** Quantification of PPARγ expression. **(L)** Quantification of p-IκB expression. **(M)** Quantification of p-NF-κB/NF-κB ratios. The results are indicated by mean ± SD. ∗*P* < 0.05, ∗∗*P* < 0.01, ∗∗∗*P* < 0.001, ∗∗∗∗*P* < 0.0001.

Western blot analysis revealed that high glucose disrupted the RAGE/NF-κB/Nrf2 pathway, increasing RAGE,p-IκB and p- NF-κB levels while reducing Nrf2 and PPARγ expression in both RSC96 and ND7/23 cells (Fig. 4A and B). GS treatment reversed these effects, decreasing RAGE (Fig. 4C) and normalizing p-IκB (Fig. 4F) and p-NF-κB (Fig. 4G) levels, while upregulating Nrf2 (Fig. 4D) and PPARγ (Fig. 4E) expression. In vivo experiments further confirmed these results, as the expression of pathway-related proteins in the sciatic nerves of diabetic rats aligned with the cellular findings. Together, these data demonstrate that GS effectively suppresses RAGE/NF-κB signaling and strengthens Nrf2/PPARγ antioxidant defense both in vitro and in vivo (Fig. 4H–M).

### 3.8 GS mitigates oxidative stress and mitochondrial dysfunction induced by high glucose

**Fig. 5:**
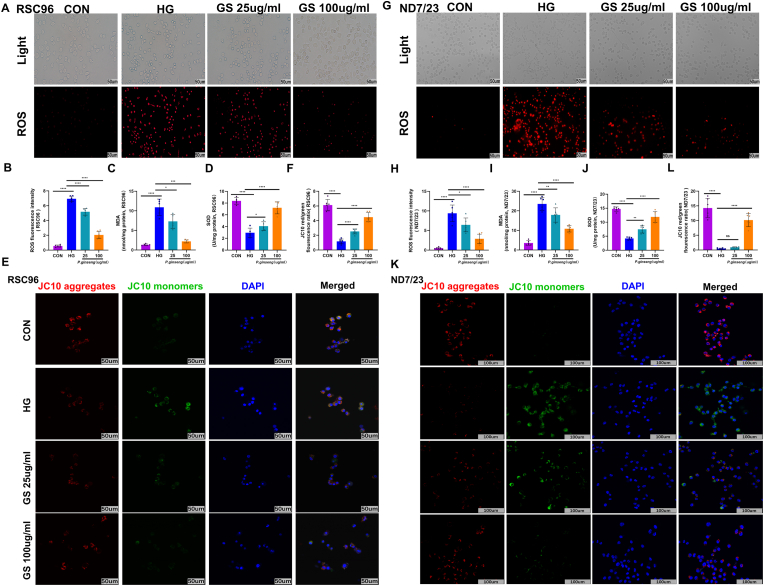
***Panax ginseng* attenuates oxidative stress and mitochondrial dysfunction in high glucose-treated RSC96 and ND7/23 cells**. **(A)** Representative fluorescence images showing ROS in RSC96 cells (100 × ; n = 6). **(B)** Quantification of fluorescence intensity of ROS. **(C)** Quantification of MDA content (n = 6). **(D)** Quantification of SOD activity (n = 6). **(E)** Representative images of JC-10 staining (200 × ; n = 6). **(F)** Red/green fluorescence ratio quantification. **(G)** Representative fluorescence images showing ROS in ND7/23 cells (100 × ; n = 6). **(H)** Quantification of fluorescence intensity of ROS. **(I)** Quantification of MDA content (n = 6). **(J)** Quantification of SOD activity (n = 6). **(K)** Representative images of JC-10 staining (200 × ; n = 6). (L) Red/green fluorescence ratio quantification. The results are indicated by mean ± SD. ∗*P* < 0.05, ∗∗*P* < 0.01, ∗∗∗*P* < 0.001, ∗∗∗∗*P* < 0.0001.

We measured intracellular ROS levels in RSC96 cells to investigate the antioxidative properties of GS. High glucose significantly increased ROS production, whereas GS treatment reduced it in a dose-dependent manner (Fig. 5A and B). Biochemical assays demonstrated that high glucose elevated MDA levels (Fig. 5C) and decreased SOD activity (Fig. 5D), both of which were normalized by GS. Mitochondrial function assessment using JC-10 staining revealed that high glucose compromised mitochondrial membrane potential (Fig. 5E and F), while GS effectively preserved it, underscoring its protective role in mitochondrial integrity. In ND7/23 neuronal cells, we observed the same effects—GS reversed high glucose-induced ROS accumulation (Fig. 5G–J) and mitochondrial dysfunction (Fig. 5K and L). Our findings demonstrate that GS exerts potent antioxidative and mitochondrial protective effects in both Schwann cells and neuronal cells.

### 3.9 GS alleviates high glucose-induced inflammatory response and apoptosis

**Fig. 6:**
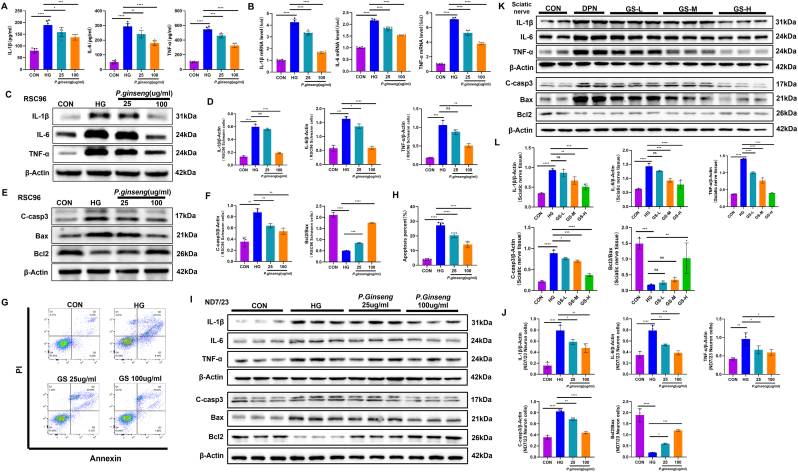
***Panax ginseng* alleviates high glucose-induced inflammatory response and apoptosis**. **(A)** Pro-inflammatory cytokine (IL-1β, IL-6, TNF-α) secretion measured by ELISA in cell culture supernatants (n = 6). **(B)** Pro-inflammatory cytokine mRNA expression levels detected by RT-qPCR (n = 6). **(C)** Pro-inflammatory cytokine protein expression of RSC96 cells analyzed by Western blot. **(D)** Quantification of IL-1β, IL-6, TNF-α (n = 3). **(E)** Representative blots of apoptosis-related protein expression in RSC96 cells analyzed by Western blot. **(F)** Quantification of apoptosis-related protein (C-casp3, Bcl2/Bax) expression (n = 3). **(G)** Apoptosis assessment by flow cytometry: Representative Annexin V-FITC/PI plots; **(H)** Quantification of apoptotic cells (n = 6). **(I)** Representative blots of pro-inflammatory cytokine and apoptosis-related protein expression in ND7/23 cells analyzed by Western blot. **(J)** Quantification of IL-1β, IL-6, TNF-α, C-casp3 and Bcl2/Bax expression (n = 3). **(K)** Representative blots of pro-inflammatory cytokine and apoptosis-related protein expression in sciatic nerves from DPN rats analyzed by Western blot. **(J)** Quantification of IL-1β, IL-6, TNF-α, C-casp3 and Bcl2/Bax expression (n = 3). The results are indicated by mean ± SD. ∗*P* < 0.05, ∗∗*P* < 0.01, ∗∗∗*P* < 0.001, ∗∗∗∗*P* < 0.0001.

ELISA analysis demonstrated that high glucose significantly increased the levels of pro-inflammatory cytokines (IL-1β, IL-6, and TNF-α), while GS treatment dose-dependently suppressed these markers (Fig. 6A). RT-qPCR results further confirmed that high glucose elevated the mRNA expression of these cytokines, which was effectively inhibited by GS (Fig. 6B). Western blot analysis revealed similar protein expression patterns in RSC96 cells (Fig. 6C and D), and flow cytometry indicated that high glucose promoted apoptosis in RSC96 cells—an effect that was mitigated by GS (Fig. 6G and H). Additionally, analysis of apoptosis-related proteins showed that high glucose upregulated pro-apoptotic Bax and downregulated anti-apoptotic Bcl-2, whereas GS restored the Bcl-2/Bax ratio and reduced caspase-3 activation (Fig. 6E and F). In ND7/23 neuronal cells, Western blot results were consistent with those in RSC96 cells—GS reversed high glucose-induced increases in inflammatory cytokines and apoptosis (Fig. 6I and J). Further in vivo experiments on the sciatic nerves of diabetic rats yielded similar findings: GS treatment reduced inflammatory protein levels and counteracted diabetes-induced alterations in apoptosis-related proteins (Fig. 6K and L). Overall, both in vitro and in vivo, and in both Schwann cells and neuronal cells, GS exhibited significant anti-inflammatory and anti-apoptotic effects.

### 3.10 Genetic and pharmacological validation of the RAGE/NF-κB/Nrf2 pathway in GS-mediated neuroprotection

**Fig. 7:**
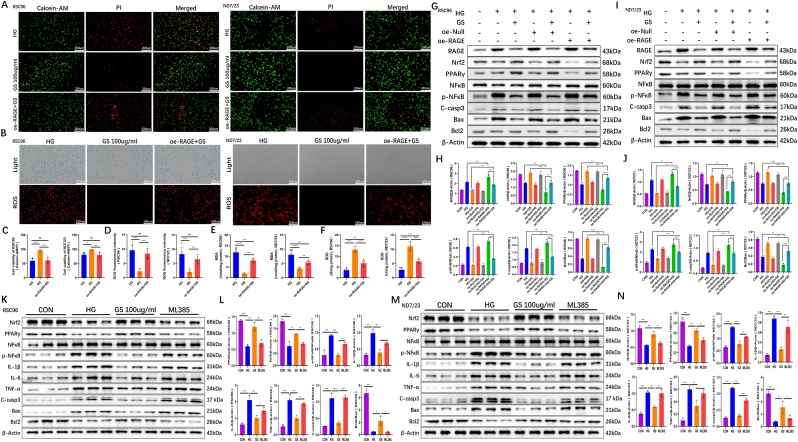
**Genetic and pharmacological validation of the RAGE/NF-κB/Nrf2 pathway in GS-mediated neuroprotection**. **(A)** Representative fluorescence images for live/dead cell staining of RSC96 and ND7/23 cells using Calcein-AM and PI (40 × ; n = 6). **(B)** Representative fluorescence images showing ROS in RSC96 and ND7/23 cells (100 × ; n = 6). **(C)** Quantitative analysis of live/dead cell staining (40 × ; n = 6). **(D)** Quantification of fluorescence intensity of ROS. **(E)** Quantification of MDA content (n = 6). **(F)** Quantification of SOD activity (n = 6). **(G)** Western blot analysis of key proteins in the RAGE/NF-κB/Nrf2 pathway and apoptosis in RSC96 cells after oe-RAGE (n = 3). **(H)** Quantification of RAGE, Nrf2, PPARγ, p-NFκB/NFκB, C-casp3, Bcl2/Bax expression in RSC96 cells. **(I)** Western blot analysis of key proteins in the RAGE/NF-κB/Nrf2 pathway and apoptosis in ND7/23 cells after oe-RAGE (n = 3). **(J)** Quantification of RAGE, Nrf2, PPARγ, p-NFκB/NFκB, C-casp3, Bcl2/Bax expression in ND7/23 cells. **(K)** Western blot analysis of key proteins in RSC96 cells after inhibiting of Nrf2 using ML385 (n = 3). **(L)** Quantification of Nrf2, PPARγ, p-NFκB/NFκB, IL-1β, IL-6, TNF-α, C-casp3, Bcl2/Bax expression in RSC96 cells. **(M)** Western blot analysis of key proteins in ND7/23 cells after inhibiting of Nrf2 using ML385 (n = 3). **(N)** Quantification of Nrf2, PPARγ, p-NFκB/NFκB, IL-1β, IL-6, TNF-α, C-casp3, Bcl2/Bax expression in ND7/23 cells.

To establish the causal role of the RAGE/NF-κB/Nrf2 pathway in the therapeutic effects of GS, we performed gain/loss-of-function experiments using RAGE overexpression (oe-RAGE) and pharmacological inhibition of Nrf2 with ML385. Live/dead staining revealed that GS treatment significantly reduced high glucose (HG)-induced cell death in both RSC96 and ND7/23 cells. However, oe-RAGE transfection abolished this protective effect, resulting in a marked increase in dead cells compared to the GS-treated group (Fig. 7A–C). Similarly, GS attenuated HG-induced ROS accumulation in RSC96 and ND7/23 cells, but oe-RAGE restored ROS levels to those observed in the HG group (Fig. 7B–D). Biochemical assays further demonstrated that oe-RAGE reversed GS-mediated improvements in oxidative stress markers, elevating MDA levels (Fig. 7E) and suppressing SOD activity (Fig. 7F) in both cell types. Western blot analysis confirmed that oe-RAGE abrogated the beneficial effects of GS on key pathway proteins. In RSC96 cells, oe-RAGE upregulated RAGE and p-NF-κB expression while downregulating Nrf2 and PPARγ (Fig. 7G). Concurrently, the anti-apoptotic Bcl-2/Bax ratio decreased, and cleaved caspase-3 (C-casp3) levels increased (Fig. 7G and H). Identical trends were observed in ND7/23 cells (Fig. 7I and J), underscoring the conserved role of RAGE across glial and neuronal compartments. Control transfection with oe-Null did not alter GS effects, confirming the specificity of RAGE-dependent modulation. To further validate the centrality of Nrf2, we treated RSC96 and ND7/23 cells with the Nrf2 inhibitor ML385 (2 μM) [19]. ML385 abolished GS-induced upregulation of Nrf2 and PPARγ and reactivated NF-κB signaling, as evidenced by elevated p-NF-κB and inflammatory cytokines (IL-1β, IL-6, TNF-α). Apoptotic markers (C-casp3, Bax) were also upregulated, while Bcl-2 declined (Fig. 7K–N). These results collectively demonstrate that GS exerts its neuroprotective effects by suppressing RAGE/NF-κB signaling and activating the Nrf2/PPARγ axis, with disruption of either arm compromising therapeutic efficacy. The consistency of these findings across Schwann cells and neuronal cells highlights the broad applicability of GS as a multitarget therapy for diabetic neuropathy.

## 4 Discussion

Diabetes mellitus and its related complications, particularly DPN, represent significant public health challenges [[20], [21], [22]]. Current treatment options for DPN are somewhat limited, mainly focusing on relieving symptoms rather than addressing the underlying causes. DPN is complex, stemming from factors such as chronic high blood sugar levels, inflammation, oxidative stress, and nerve cell death, which calls for a comprehensive treatment approach. Research has shown that pro-inflammatory cytokines and oxidative stress markers can worsen nerve damage [[23], [24], [25]]. Our study highlights the potential of GS as a multi-target therapeutic agent for DPN. The results demonstrate that GS not only improves peripheral nerve function in DPN models but also enhances cell survival and movement in hyperglycemic conditions. Furthermore, using network pharmacology, we explored the complex interactions between the active compounds in GS and their targets, revealing how GS exerts its protective effects on nerves. This research contributes to the growing body of evidence supporting the integration of herbal medicines into modern treatment strategies, indicating a promising direction for developing effective therapies for 10.13039/501100017234DPN. The implications of our findings extend beyond immediate clinical use and may guide future research in managing diabetes-related complications.

*Panax ginseng* is a well-known medicinal herb celebrated for its wide range of health benefits, especially in managing diabetes and its related complications. The key active components of GS include ginsenosides, volatile oils, polysaccharides, and amino acids, all of which demonstrate significant biological effects in different physiological and pathological conditions [26,27]. A notable clinical trial, which was randomized, double-blind, and placebo-controlled, explored the effects of Korean red ginseng (KRG) on peripheral neuropathy in patients with type 2 diabetes. The results indicated that a 24-week course of KRG supplementation significantly improved current perception thresholds at all tested frequencies in the lower limbs. This improvement was particularly pronounced in patients who had been living with diabetes for a longer period or who had pre-existing neuropathy at the beginning of the study [28]. Our research indicates that GS significantly alters the characteristics of DPN by reversing key pathological changes. The analysis showed that GS improved mechanical pain thresholds and thermal response times in diabetic rats induced by STZ, suggesting its potential to alleviate the sensory deficits commonly associated with DPN. Additionally, the observed improvements in morphology, such as the restoration of myelin sheath integrity and axonal structure, emphasize GS's role in promoting nerve health in diabetic conditions. The significant reduction in abnormal axons and the enhancement of the G-ratio following GS treatment indicate a protective effect against degeneration typically seen in DPN. This restoration of morphology is crucial, as it is linked to the alleviation of the phenotypic symptoms of neuropathy. Moreover, the increased expression of MBP and NF-H after GS treatment further highlights its importance in maintaining myelin integrity and supporting axonal structures. These molecular improvements play a significant role in normalizing neural function, demonstrating a direct relationship between GS administration and the reduction of DPN symptoms. In conclusion, the combined data suggest that GS not only reduces the functional impairments associated with DPN but also fosters significant morphological and molecular enhancements in diabetic nerves, indicating its substantial therapeutic potential in addressing this debilitating condition.

The mechanisms behind the RAGE/NF-κB signaling pathway in DPN are not yet fully understood. When glucose levels rise, the receptor for advanced glycation end-products (RAGE) is upregulated, which activates inflammatory signaling through the NF-κB pathway [29]. This process is known to increase the production of pro-inflammatory cytokines like TNF-α and IL-6, which can worsen neuronal damage [30]. Research shows that certain components of GS, especially ginsenosides Rb1 and Rg3, can inhibit the activation of NF-κB, leading to a decrease in the release of these harmful cytokines [31,32]. Our findings support this, indicating that GS treatment reduces the overactivity of this pathway through two main actions: lowering RAGE expression and inhibiting the phosphorylation of IκB. Together, these effects highlight the strong anti-inflammatory and neuroprotective properties of GS. These results align with previous studies that have documented the antioxidant and anti-inflammatory benefits of GS [[33], [34], [35], [36]]. The antioxidant potential of GS plays a significant role in influencing the NF-κB pathway by enhancing the expression of protective factors through the Nrf2/PPARγ signaling route [37]. Under normal physiological conditions, Nrf2 moves to the nucleus, where it activates the transcription of genes that encode antioxidant enzymes, helping to counteract oxidative damage [38]. However, elevated glucose levels associated with diabetes can impair Nrf2 activity, leading to oxidative stress and subsequent neuronal apoptosis. Research indicates that GS can restore the levels of Nrf2 and PPARγ, thereby strengthening oxidative defense mechanisms and enhancing neuronal protection. Moreover, GS also affects other signaling pathways related to apoptosis and cellular survival [39], effectively reducing levels of apoptosis-related proteins like caspase-3, which reinforces its role in promoting neuronal survival in the context of DPN [40]. By modulating these various pathways, GS alleviates the symptoms of 10.13039/501100017234DPN and supports the regeneration and repair of damaged nerve structures, showcasing its multi-target therapeutic efficacy. These findings suggest that GS operates through interconnected biological pathways, highlighting its potential as a comprehensive treatment option for DPN that addresses both inflammatory and oxidative stress factors.

The Protein Kinase C (PKC) family plays a pivotal role in diabetic peripheral neuropathy (DPN) by mediating oxidative stress and neuroinflammation. PKC activation stimulates NADPH oxidase, increasing reactive oxygen species (ROS) production, which damages cellular components and induces neuronal apoptosis [41]. This oxidative stress is exacerbated by PKC-dependent mitochondrial dysfunction, impairing energy metabolism and creating a vicious cycle of ROS generation [42]. Concurrently, PKC dysregulates antioxidant defenses (e.g., superoxide dismutase), further perpetuating oxidative damage [43]. PKC also drives neuroinflammation via PKC-NFκB signaling, elevating pro-inflammatory cytokines and activating glial cells, which exacerbate neuronal injury and pain pathways [44,45]. Together, these mechanisms highlight PKC as a critical therapeutic target for mitigating DPN progression. Emerging evidence suggests that Panax ginseng extracts may exert therapeutic effects on peripheral neuropathy through modulation of PKC signaling pathways. Specifically, PKCδ has been implicated in inflammatory responses in mouse keratinocytes, where its activation triggers GM-CSF release. Notably, ginsenoside Rg3 has been shown to suppress GM-CSF production by inhibiting PKCδ and ERK phosphorylation [46]. Conversely, ginsenoside Rb1 promotes neural regeneration through PKCε activation, demonstrating its neuroprotective potential. Studies indicate that Rb1 enhances CREB phosphorylation, a critical transcription factor regulating nerve growth factor expression, thereby supporting neuronal survival and regeneration [47]. In our study, network pharmacology analysis prioritized investigation of the RAGE/NFκB pathway, while PKC-related pathways were not identified as primary targets in our predictive model. However, existing literature clearly demonstrates that Panax ginseng and its bioactive components can differentially modulate various PKC isoforms, suggesting this pathway warrants further exploration in future mechanistic studies of diabetic peripheral neuropathy.

This study highlights the significant therapeutic benefits of GS for DPN by improving peripheral nerve function and cellular health in diabetes-related conditions. Through network pharmacology, the research explores how GS's bioactive components interact with various biological targets, focusing on their roles in reducing inflammation, oxidative stress, and cell death. By clarifying the mechanisms through which GS operates, this research adds valuable insights into alternative treatment strategies for 10.13039/501100017234DPN and supports the clinical use of GS as a promising option to enhance patient outcomes in managing this condition. However, the study does have several limitations. The use of animal models, particularly STZ-induced diabetic rats, does not fully capture the complex pathophysiology of human DPN due to biological differences. Additionally, while network pharmacology provides insights into multi-target interactions, the lack of comprehensive validation through clinical trials restricts the broader applicability of the findings. Future research should focus on addressing these limitations by incorporating human cellular models and clinical data to improve the relevance of the results.

## Author contribution

**Pengcheng Liu:** Conceptualization, Methodology, Validation, Writing – original draft. **Jie Zhang:** Investigation, Methodology, Visualization. **Teng Yang:** Investigation, Methodology, Formal analysis. **Bang Su:** Methodology, Validation. **Qidong Shi:** Investigation, Methodology. **Yao Song:** Methodology, Formal analysis. **Xin Yu:** Conceptualization, Funding acquisition, Methodology, Project administration, Supervision, Validation.

## Declaration of competing interest

The authors declare that the study was performed without any commercial conflict.
